# Diet and physical activity in multiple sclerosis: associations with quality of life and disability by disease duration

**DOI:** 10.1007/s10072-026-09352-2

**Published:** 2026-10-20

**Authors:** Nupur Nag, Maggie Yu, Cristina A.F. Román, Annette Vaglio-Garro, Hollie Schmidt

**Affiliations:** 1 Neuroepidemiology Unit, Centre of Epidemiology and Biostatistics, Melbourne School of Population and Global Health, The University of Melbourne, Melbourne, VIC Australia; 2 Keck School of Medicine, University of Southern California, Los Angeles, CA USA; 3 Kessler Foundation, West Orange, NJ USA; 4 Ludwig Boltzmann Institute for Traumatology, The Research Center in Cooperation with AUVA, Vienna, Austria; 5 Austrian Cluster for Tissue Regeneration, Vienna, Austria; 6 Accelerated Cure Project for Multiple Sclerosis, Waltham, MA USA

**Keywords:** Multiple sclerosis, Lifestyle, Observational study, Health outcomes, Self-management

## Abstract

**Background:**

Healthy lifestyle factors are associated with higher quality of life (QoL) and reduced disability in people with multiple sclerosis (pwMS). Whether these associations differ according to MS duration is unclear. We examined associations between diet, supplement use, wellness activities, and physical activity with QoL and ambulatory disability, and whether these associations differed between pwMS diagnosed within ≤ 5 years versus > 5 years.

**Methods:**

Cross-sectional self-reported survey data from 1,083 pwMS were analysed. Diet, supplement use, and wellness activities were queried via tick-box options and recategorised. Physical activity was assessed using the Godin–Shephard Leisure-Time Physical Activity Questionnaire. QoL was measured using Neuro-QoL, and ambulatory disability using the Patient Determined Disease Steps. Multivariable linear and moderation analyses were conducted.

**Results:**

Associations differed by MS duration: a low-saturated fat diet and “other” diets were associated with lower QoL in the > 5-years group, whereas omega-3 supplement use was associated with higher physical QoL in the ≤ 5-years group. Physical activity was associated with higher QoL in both groups, with mostly stronger associations within ≤ 5-years. Very active physical activity was associated with lower ambulatory disability across both groups. Mind-based wellness activities were associated with lower QoL in the > 5-years group, with no group differences. Vitamin D supplement use was not associated with outcomes.

**Conclusions:**

Physical activity is associated with higher QoL and lower disability irrespective of MS duration and should be integrated across the disease course. MS duration may modify associations between other lifestyle factors and QoL, supporting consideration of disease stage when tailoring lifestyle recommendations.

**Supplementary Information:**

The online version contains supplementary material available at https://doi.org/10.1007/s10072-026-09352-2.

## Introduction

Multiple sclerosis (MS) is a progressive neuroinflammatory disorder that affects more than 2.8 million people worldwide [1]. Individuals are typically diagnosed between the ages of 20–40, interrupting a crucial time of educational and career progress, family planning, and the overall growth of financial, functional, and personal independence [2]. These, combined with symptoms of MS, including fatigue, pain, mobility limitations, and cognitive impairment significantly contribute to reduced quality of life (QoL) [3, 4]. MS is classified as four phenotypes: relapsing-remitting MS, primary progressive MS, secondary progressive MS, and clinically isolated syndrome; from 2013, disease course is further characterised by time-framed modifiers of activity and progression [5, 6]. Disability worsening can occur even in relapsing MS independent of overt relapses, suggesting continuous view of inflammatory activity and progression biology [7]. As disease burden may increase over time, and treatment options for progressive MS remain limited [8], symptom management via lifestyle modifications are of growing interest.

Lifestyle modifications through diet, physical activity, dietary supplement use, and stress management, are associated with better health outcomes in pwMS [9, 10]. Several diets, including low-saturated fat and anti-inflammatory, have been associated with reduced ambulatory disability and improved QoL [11, 12], with commonality being reduced consumption of processed foods. Evidence for the benefits of supplement use is mixed; some studies show associations between omega-3 and vitamin D with QoL, relapse rate, and inflammatory markers, while others report no effect [13, 14]. Wellness practices of yoga, mindfulness, and meditation, may improve mental health and social participation, while their impact on level of ambulatory disability is unclear [15, 16]. Lifestyle modifications are often adopted together as part of self-management strategies in MS with studies showing cumulative effects on QoL and clinical outcomes [17, 18]. Accordingly, examining multiple lifestyle factors may provide a more comprehensive understanding of lifestyle influences on MS outcomes. While lifestyle factors are increasingly recognised as important adjuncts to MS management, it remains unclear whether their associations with QoL and disability differ according to disease duration. Identifying whether lifestyle benefits are time-dependent is critical for optimising when and how lifestyle strategies are introduced in clinical care. Lifestyle modifications early in the disease course may have stronger effects on health outcomes due to greater neuroplasticity and less accumulated disability [19]. This is also observed with pharmacological treatments, where early initiation leads to better long-term outcomes [20].

The present study aimed to examine associations between lifestyle factors (diet, supplement use, wellness activities, and physical activity), with QoL as a primary outcome and ambulatory disability as a secondary outcome. We further investigated whether these associations differ according to MS duration, comparing individuals diagnosed within five years or less - a period commonly used to represent early MS and during which treatment responsiveness and functional reserve are greatest - with those with a diagnosis of more than five years. We hypothesised that healthy lifestyle factors would be associated with higher QoL and lower disability, and that associations may be stronger earlier in the disease course. Given the cross-sectional design and the range of lifestyle factors and outcomes examined, this study was exploratory. These findings may help inform MS care by highlighting when lifestyle modifications may be most relevant across different stages of disease progression. 

## Methods

Ethics approval was obtained from the University of Melbourne (ID #1956113.1). This study adheres to the Strengthening the Reporting of Observational Studies in Epidemiology (STROBE) guidelines (Supplementary Table 1).

### Study design and participants

Recruitment to the REAL MS study commenced in 2014, with participation open to pwMS and the general population aged ≥ 21 years. Consenting participants voluntarily complete optional online surveys on demographics, health, and lifestyle, at 6-month intervals. Baseline data from participants registered between November 2014 to July 2020 (*n* = 3374) was extracted then restricted for analysis to participants reporting a clinician-confirmed MS diagnosis, confidence in MS diagnosis, and with complete data for date of diagnosis, diet, wellness, physical activity, QoL, and ambulatory disability. Only relapsing-remitting MS (non-progressive), primary progressive MS, and secondary progressive MS phenotypes were included. The final study population thus included 1,083 pwMS (Supplementary Fig. 1).

### Data collection and measurement

#### Demographics and clinical outcomes

Demographics queried and recategorised included age, sex, education, relationship status, employment status, country of birth, and annual household income, as detailed previously [21]. MS duration was calculated by year of survey completion minus year of diagnosis, then dichotomised to ≤ 5-years and > 5-years MS duration. MS phenotype was categorised to non-progressive vs. progressive. Symptoms experienced prior to diagnosis was queried by the question: “Before your first exacerbation or diagnosis, did you experience any symptoms that you feel were due to MS but did not meet the definition of a relapse or a doctor did not consider these symptoms consistent with MS?” and responses dichotomised to (Yes versus No/not sure). Body mass index (BMI) was calculated by weight (kg)/height (m)^2^, then classified as underweight/normal, overweight and obese [22].

#### Assessment of lifestyle factors

Twenty-three diet options, 25 wellness activities, and 29 supplements were individually queried via tick-box options of “used” and “used and helpful” in the past 6 months to improve wellbeing. Participants could select multiple lifestyle options; subcategories were not mutually exclusive. Responses of “used” and “used and helpful” were combined to indicate engagement with the practice. These were dichotomised to Yes = used/used and helpful versus No = none selected. Separate analyses for ‘used’ vs. ‘used and helpful’ were not performed as the ‘used only’ category included < 10% of participants. Diet and wellness variables were re-categorised (Table 1) and analysed as binary (Yes = used/used and helpful of ≥ 1 option within category). Of 29 supplements, only vitamin D and omega-3 were included in this analysis due to their associations with improved outcomes in pwMS [23–25].

Physical activity was assessed via the Godin-Shephard Leisure-Time Physical Activity Questionnaire, where participants self-report frequency (0–7 days) of strenuous, moderate, and mild exercise for ≥ 15 min during their leisure time in the preceding seven days [26]. Total leisure activity score was calculated as (9×strenuous) + (5×moderate) + (3×light) and classified as less active (< 14), moderately active (14–23), and very active (≥ 24).

**Table 1:** Lifestyle categories

Lifestyle	Category	Inclusions
Diet	Anti-inflammatory	Anti-inflammatory, fasting/calorie restriction, gluten-free, Mediterranean
	Low-saturated fats	Jelinek, Swank, low-fat, ovo-lactovegetarian, vegetarian, vegan, lacto-vegetarian, Ornish, Pritikin, pescatarian
	Low-carbohydrate	Atkins, ketogenic, paleo, Wahls, low-carbohydrate
	Other	Organic, low sodium, low sugar, semi-vegetarian
Supplements	Vitamin D	Vitamin D
	Omega-3	Omega-3, DHA or EPA fatty acid, fish-oil, flaxseed/flaxseed oil
Wellness	Mind	Meditation, mindfulness, guided imagery, stress management, biofeedback, brain training, cognitive behavioural therapy
	Mind-body	Tai chi, yoga, qigong, exercise therapy, relaxation exercise, deep breathing exercises, progressive relaxation
	Body	Acupuncture, ayurveda, chiropractic/osteopathic manipulation, craniosacral therapy, energy healing, hypnosis, massage, reflexology
	Other	Chelation therapy, naturopathy, traditional healing
Physical activity	Less active Moderately active Very active	leisure activity score < 14 leisure activity score 14–23 leisure activity score > 23

#### Health outcomes

QoL, the primary outcome, was assessed via the NeuroQoL Adult Short Form [27], comprising physical, mental, and social QoL domains. Each subdomain score was based on 5–9 items rated on a 5-point Likert scale. Item responses were summed and converted to T-scores on a common metric (mean = 50, SD = 10), anchored to the reference population specified for each Neuro-QoL item bank. For mobility, fine motor, anxiety, depression, positive affect, cognitive function, social participation, and social satisfaction subdomains, T-scores are derived from normative data gathered from a subset of the U.S. population without neurological disorders; and for fatigue, sleep disturbance, emotional dyscontrol, and stigma subdomains, T-scores are derived from normative data derived from a sample of individuals with a diagnosed neurological disorder (e.g., MS, epilepsy, stroke, amyotrophic lateral sclerosis, or Parkinson’s disease). Higher T-scores equate to higher measured concepts across all variables; higher scores indicate worse health for symptom domains (e.g., fatigue, sleep disturbance, anxiety/depression, stigma) and better health for function/well-being domains (e.g., mobility, fine motor function, cognitive function, and social participation/satisfaction). Communication short form does not have an item response theory (IRT)–based T-score; therefore, communication was analysed and reported using the manual-recommended scale derived from the summed raw score (0–100 metric).

Ambulatory disability, examined as secondary outcome, was queried via the Patient Determined Disease Steps (PDDS), and scores consolidated to low (0–2), moderate (3–5) and high (6–8) disability [28].

### Statistical analysis

Baseline characteristics were summarised by MS duration group (≤ 5-years and > 5-years) using means (standard deviation) for continuous variables and numbers (percentages) for categorical variables.

Lifestyle factors (diet, supplement use, wellness activities, and physical activity) were modelled as separate exposures and examined in parallel rather than hierarchically because prior literature suggests that multiple lifestyle domains may influence MS outcomes.

#### Primary analysis

The primary objective was to examine associations between lifestyle factors and QoL, and whether these associations differed by MS duration (≤ 5-years and > 5-years). Associations with QoL domains were assessed using multiple linear regression models, estimating regression coefficients (β) and 95% confidence intervals (CIs). Models were adjusted for age, sex, education, BMI, symptoms before diagnosis, MS phenotype, and ambulatory disability. Effect modification by MS duration was assessed by including an interaction term between MS duration and each lifestyle factor in all models. Differences between MS duration groups were evaluated based on the statistical significance of the interaction term.

#### Secondary analysis

The secondary objective was to examine associations between lifestyle factors and ambulatory disability. Ambulatory disability severity (low, moderate, and high) categories were analysed using multinomial regression, estimating prevalence ratios (PRs) and 95% CIs for moderate and high disability relative to low disability. This approach was chosen to estimate category-specific associations and PRs, which were considered more interpretable in this cross-sectional context than cumulative odds ratios from an ordinal proportional-odds model. Models were adjusted for age, sex, education, BMI, symptoms before diagnosis, and MS phenotype.

#### Multiplicity and statistical considerations

Because multiple lifestyle factors and QoL domains were examined, analyses were considered exploratory and results were interpreted with caution, taking consideration of potential multiplicity. As Neuro-QoL was designed to assess conceptually distinct QoL domains with limited overlap, multiplicity was addressed within domains rather than across all outcomes combined. As a sensitivity analysis, Bonferroni correction was applied within each domain by adjusting the significance threshold according to the number of outcomes tested to control the family-wise error rate [29] (e.g., α = 0·0125 for four outcomes in the physical QoL domain and α = 0·025 for two outcomes in the social QoL domain). Greater emphasis was placed on the consistency and overall pattern of associations across domains than on isolated p-values.

Data analyses were conducted in Stata/SE 18.0 (StataCorp, College Station, TX).

## Results

### Participant characteristics

The study included 1,083 participants, with baseline characteristics categorised by MS duration (Table 2). Participants were grouped into ≤ 5-years MS duration (26.3%) and > 5-years MS duration (73.7%) groups. Most participants were female, born in the United States, university educated, partnered, and had non-progressive phenotype. Compared to pwMS with > 5-years MS duration, those with ≤ 5-years MS duration were younger (45 vs. 56 years) and differed in time since diagnosis (3 vs. 15 years) and time since symptom onset (7 vs. 20 years). PwMS with ≤ 5-year MS duration were more likely to be born outside of the USA (24% vs. 12%), employed (64% vs. 38%), reported higher household incomes (≥$100,001: 39% vs. 30%), and were less likely to have progressive MS (21% vs. 37%) or severe ambulatory disability (4% vs. 22%). They were also more likely to have used an anti-inflammatory diet (42% vs. 34%), a low-saturated fat diet (23% vs. 17%), have engaged in mind activities (67% vs. 59%), and been physically active (55% vs. 41%).

**Table 2:** Characteristics and lifestyle of study participants with ≤5-years vs >5-years MS duration

Characteristics	MS Duration			MS Duration					
	≤ 5-years *N* = 285	> 5-years *N* = 798		Lifestyle	≤ 5-years *N* = 285	> 5-years *N* = 798			
	**n (%)**		***p***		**n (%)**				***p***
**Sex**				**Diet**					
Male	58 (20)	178 (22)	Ref.	Anti-inflammatory					
Female	227 (80)	617 (78)	0.464	Not used	166 (58)	529 (66)			Ref.
**Country of birth**				Used/helpful	**119 (42)**	**269 (34)**			**< 0.05**
USA	216 (76)	705 (89)	Ref.	Low saturated fat					
Other	**69 (24)**	**92 (12)**	**< 0.001**	Not used	220 (77)	662 (83)			Ref.
**University degree**				Used/helpful	**65 (23)**	**136 (17)**			**< 0.05**
Yes	198 (70)	556 (70)	Ref.	Low carbohydrate					
No	87 (30)	240 (30)	0.906	Not used	215 (75.4)	623 (78)			Ref.
**Partnered**				Used/helpful	70 (24.6)	175 (22)			0.377
Yes	198 (70)	585 (74)	Ref.	Other					
No	86 (30)	209 (26)	0.214	Not used	184 (65)	555 (70)			Ref.
**Paid employment**				Used/helpful	101 (35)	243 (30)			0.133
Yes	182 (64)	302 (38)	Ref.	**Supplements**					
No	**102 (36)**	**486 (62)**	**< 0.001**	Vitamin D					
**Household income (USD)**				Not used	37 (13)	129 (16)			Ref.
≤ $ 50,000	59 (32)	147 (39)	Ref.	Used/helpful	248 (87)	669 (84)			0.172
$50,001-100,000	52 (28)	116 (31)	0.628	Omega-3					
≥ $100,001	**72 (39)**	**112 (30)**	**< 0.05**	Not used	165 (58)	492 (62)			Ref.
**BMI**				Used/helpful	120 (42)	306 (38)			0.271
Under/healthy	137 (48)	350 (44)	Ref.	**Wellness activities**					
Overweight	72 (25)	227 (29)	0.204	Mind					
Obese	76 (27)	216 (27)	0.520	Not used	85 (33)	277 (41)			Ref.
**Disability (PDDS)**				Used/helpful	**172 (67)**	**395 (59)**			**< 0.05**
Low	173 (61)	299 (38)	Ref.	Mind-body					
Moderate	**101 (35)**	**326 (41)**	**< 0.001**	Not used	109 (42)	275 (41)			Ref.
Severe	**11 (4)**	**172 (22)**	**< 0.001**	Used/helpful	148 (58)	397 (59)			0.524
**MS phenotype**				Body					
Non-progressive	226 (79)	504 (63)	Ref.	Not used	130 (51)	346 (52)			Ref.
Progressive	**59 (21)**	**294 (37)**	**< 0.001**	Used/helpful	127 (49)	326 (49)			0.270
**Symptoms pre diagnosis**				Other activities					
Yes	168 (65)	451 (61)	Ref.	Not used	250 (97)	653 (97)			Ref.
No/unsure	92 (35)	284 (39)	0.347	Used/helpful	7 (3)	19 (3)			0.459
	**Median (IQR)**		**p-value**	**Physical activity**					
Age, y	**45 (37–53)**	**56 (49–63)**	**<0.001**	Less active	82 (29)	287 (36)			Ref.
Duration since diagnosis, y	**3 (1–4)**	**15 (10–22)**	**< 0.001**	Moderate	46 (16)	184 (23)			0.514
Duration since symptom onset, y	**7 (2–12)**	**20 (9–29)**	**< 0.001**	Very active	**157 (55)**	**327 (41)**			**< 0.001**

Abbreviations: *BMI* body mass index, *PDDS* patient determined disease steps, *Ref* reference, *SD* standard deviation, *USA* United States of America, *USD* United States Dollar. P values indicate statistical differences between **≤ **5-years and > 5-years MS duration. Bolded values indicate significant group difference (*p*<0.05)

Across QoL outcomes, pwMS with > 5-years MS duration had T-scores of more than 0.5 SD below the U.S. population norms for mobility, fine motor skills, and social satisfaction (Table 3). This threshold is based on established guidance suggesting that a difference of approximately 0.5 SD (≈ 5 Neuro-QoL T-score points) reflects a meaningful difference relative to population norms for patient-reported outcome measures [27]. Compared to pwMS with > 5-years MS duration, those with ≤ 5-years MS duration had better scores for mobility, fine motor function, social satisfaction, and social participation, but higher anxiety and emotional dyscontrol scores and less ambulatory disability.

**Table 3:** QoL and disability of participants with ≤ 5-years versus >5-years MS duration

	MS Duration	
	≤ 5-years *n* = 285	> 5-years *n* = 798
Physical QoL		
Mobility	48.7 (9.2)	43.4 (9.4)***
Fine motor	46.3 (8.7)	44.1 (9.3)***
Fatigue	51.9 (9.3)	52.3 (9.2)
Sleep disturbance	54.1 (8.5)	53.5 (8.7)
Mental QoL		
Anxiety	52.3 (7.7)	51.1 (8.4)*
Depression	48.2 (8.1)	47.9 (8.1)
Positive affect	51.9 (8.3)	52.1 (8.2)
Emotional dyscontrol	49.4 (9.5)	48.0 (9.6)*
Stigma	49.7 (8.0)	50.3 (7.6)
Cognitive function	45.9 (10.7)	46.2 (10.4)
Communication ^a^	22.0 (3.5)	22.1 (3.4)
Social QoL		
Participation	46.7 (8.0)	45.5 (7.3)*
Satisfaction	45.1 (7.4)	43.9 (6.5)*
**Disability (PDDS)**		
Normal/mild	173 (61)	299 (38)
Moderate	101 (35)	326 (41)***
Severe	11 (4)	172 (22)***

^a^Total raw score. Bolded mean scores indicate differences > 5 points on the QoL T-scale metric (0.5 SD) than the clinical or US general population. SD= standard deviation. Significant group differences: *p < 0.05, **p < 0.01, ***p < 0.001. Asterisks in the >5-years column denote differences between ≤ 5-years and >5-years MS duration

### Associations between lifestyle and QoL (primary outcome)

Associations between lifestyle factors and QoL varied across domains, with limited evidence of effect modification by MS duration based on interaction tests (Tables 4-6). Overall, physical activity showed the most consistent and robust associations with QoL, whereas findings for diet, supplement use, and wellness activities were less consistent. Diet showed variable associations with QoL domains (Table 4). Anti-inflammatory and low-carbohydrate diets were associated with higher stigma scores in pwMS with > 5-years group, although there was no evidence of interaction by MS duration. A low-saturated fat diet showed evidence of effect modification by MS duration for mobility (*P*_interaction_ = 0.002) and stigma (*P*_interaction_ = 0.007), with opposite directions of association observed between duration groups. In addition, a low-saturated fat diet was associated with higher anxiety scores in the >5-years group only, with no evidence of interaction. The ‘other’ diet category showed evidence of effect modification by MS duration for fine motor (*P*_interaction_ = 0.022), communication (*P*_interaction_ = 0.013), and stigma (*P*_interaction_ = 0.020), with associations observed primarily in the > 5-years group. It was also associated with lower cognitive function in the > 5-years group, although the interaction test was not significant. After Bonferroni correction, associations for low-saturated fat diet and the ‘other’ diet category in the > 5-years group remained significant.

Mind activities showed no evidence of associations with QoL scores in pwMS with ≤ 5-years MS duration (Table 5). In pwMS with > 5-years MS duration, mind activities corresponded to lower QoL scores across several domains and remained significant after Bonferroni correction, with no evidence of MS duration group differences. Mind–body, and other wellness activities showed no evidence of associations with QoL in either group. Body activities were associated with lower anxiety scores in pwMS with ≤5-years MS duration and lower depression scores in those with >5-years MS duration, although neither association remained significant after Bonferroni correction.

Vitamin D supplement use showed no evidence of associations with QoL scores in either MS duration group (Table 6). Omega-3 supplement use corresponded to higher mobility, fine motor, and social satisfaction scores and lower sleep disturbance scores in the ≤ 5-years group. However, these did not remain significant after Bonferroni correction. No associations were observed for omega-3 supplement use among pwMS with > 5-years MS duration. Group differences were significant for mobility (*P*_interaction_ =0.045), fine motor (*P*_interaction_= 0.015), and sleep disturbance (*P*_interaction_ =0.018).

Physical activity was consistently associated with higher QoL across multiple domains in both MS duration groups (Table 6). Moderate physical activity showed positive associations across several QoL domains among pwMS with >5-years MS duration, although there was no evidence of interaction by MS duration. Very active physical activity was associated with higher QoL scores across physical, mental, and social domains, with several associations remaining after Bonferroni correction. There was evidence of interaction for selected outcomes, including depression (*P*_interaction_ = 0.031), positive affect (*P*_interaction_ = 0.032), communication (*P*_interaction_ = 0.026), social participation (*P*_interaction_ = 0.019), and social satisfaction (*P*_interaction_ = 0.010), suggesting differences in associations by MS duration.

**Table 4:**
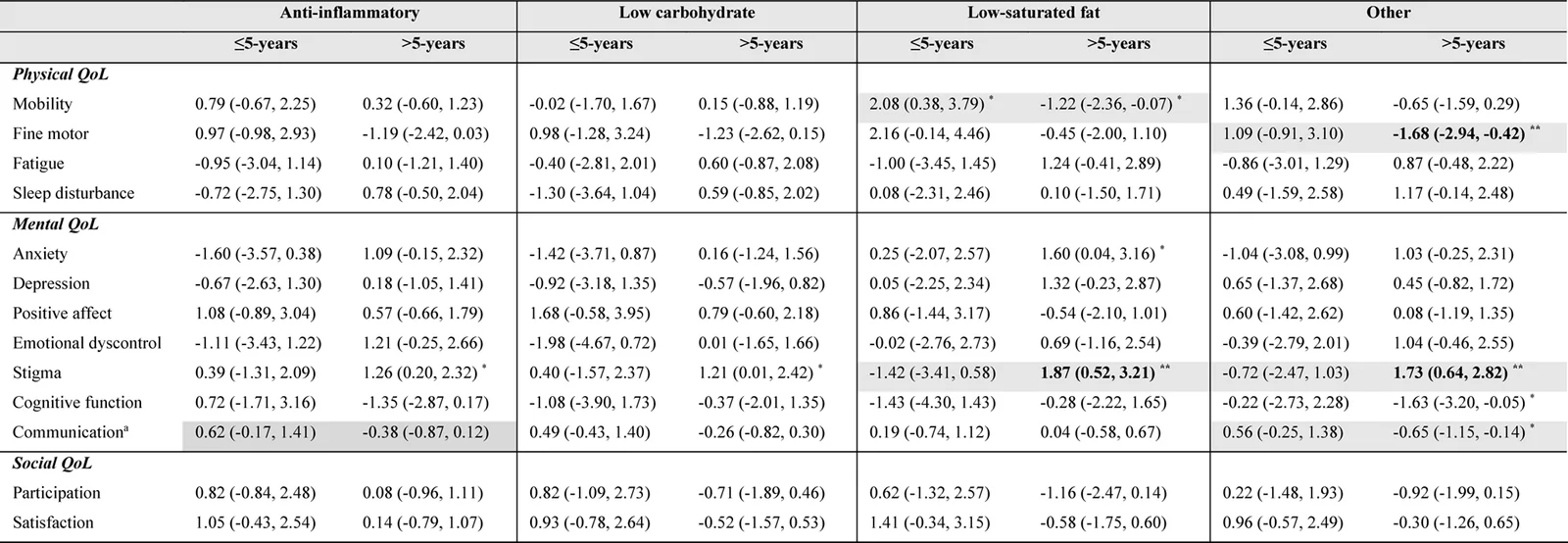
Associations between diet and QoL subdomains, in ≤ 5-years vs. > 5-years MS duration

*QoL* quality of life. Values are adjusted regression coefficients (β) with 95% confidence intervals from multivariable linear regression models adjusted for age, sex, body mass index, education, ambulatory disability, symptoms before diagnosis, and MS phenotype. Asterisks indicate nominal, unadjusted p values (*p<0.05; **p<0.01; ***p<0.001). Bold indicates associations meeting the Bonferroni-corrected significance threshold within each QoL domain (physical QoL: *p* < 0.0125 [0.05/4]; mental QoL: *p* < 0.007 [0.05/7]; social QoL: *p* < 0.025 [0.05/2]). Shaded cells indicate evidence of effect modification by MS duration (≤5-years vs >5-years) (p for interaction <0.05). ^a^ Total raw score

**Table 5:**
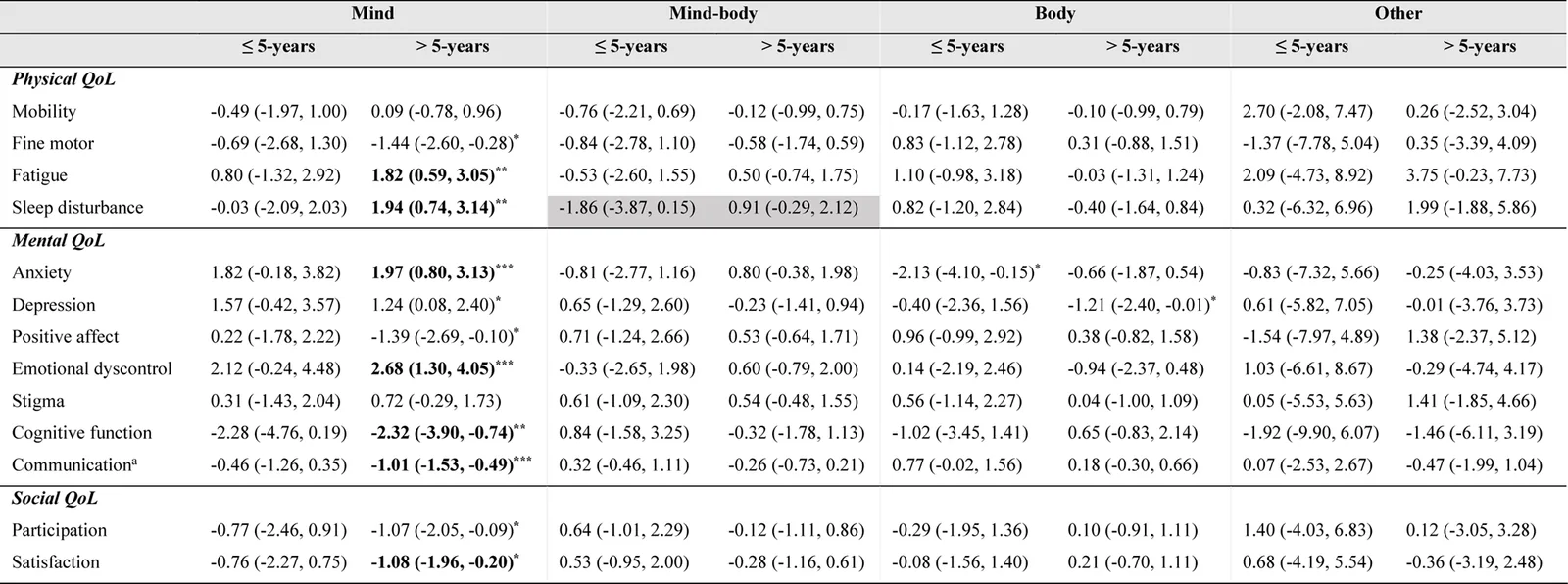
Associations between wellness activities and QoL domains, in ≤  5-years vs. >  5-years MS duration

*QoL* quality of life. Values are adjusted regression coefficients (β) with 95% confidence intervals from multivariable linear regression models adjusted for age, sex, body mass index, education, ambulatory disability, symptoms before diagnosis, and MS phenotype. Asterisks indicate nominal, unadjusted p values (*p<0.05; **p<0.01; ***p<0.001). Bold indicates associations meeting the Bonferroni-corrected significance threshold within each QoL domain (physical QoL: *p* < 0.0125 [0.05/4]; mental QoL: *p* < 0.007 [0.05/7]; social QoL: *p* < 0.025 [0.05/2]). Shaded cells indicate evidence of effect modification by MS duration (≤5-years vs >5-years) (p for interaction <0.05). ^a^ Total raw score

**Table 6:**
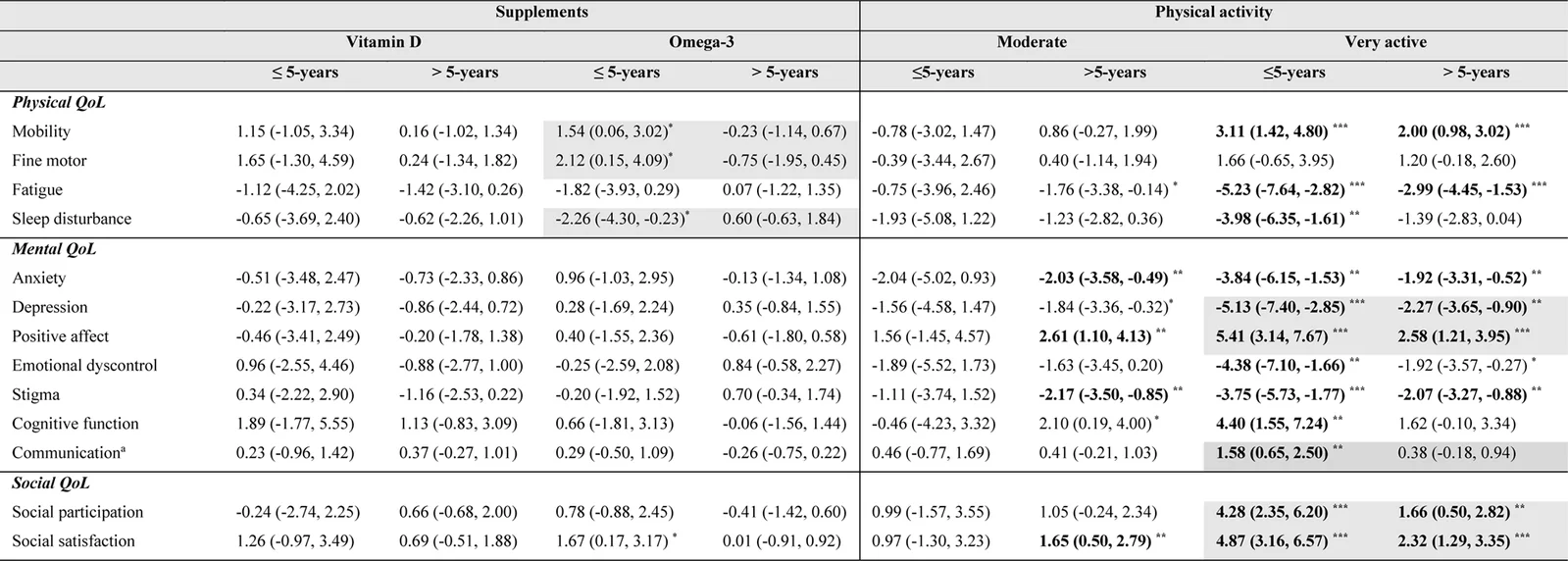
Associations between supplement use, physical activity and QoL subdomains, in ≤ 5-years vs. > 5-years MS duration

*QoL* quality of life. Values are adjusted regression coefficients (β) with 95% confidence intervals from multivariable linear regression models adjusted for age, sex, body mass index, education, ambulatory disability, symptoms before diagnosis, and MS phenotype. Asterisks indicate nominal, unadjusted p values (*p<0.05; **p<0.01; ***p<0.001). Bold indicates associations meeting the Bonferroni-corrected significance threshold within each QoL domain (physical QoL: *p* < 0.0125 [0.05/4]; mental QoL: *p* < 0.007 [0.05/7]; social QoL: *p* < 0.025 [0.05/2]). Shaded cells indicate evidence of effect modification by MS duration (≤5-years vs >5-years) (p for interaction <0.05). ^a^ Total raw score

### Associations between lifestyle and ambulatory disability (secondary outcome)

**Fig. 1:**
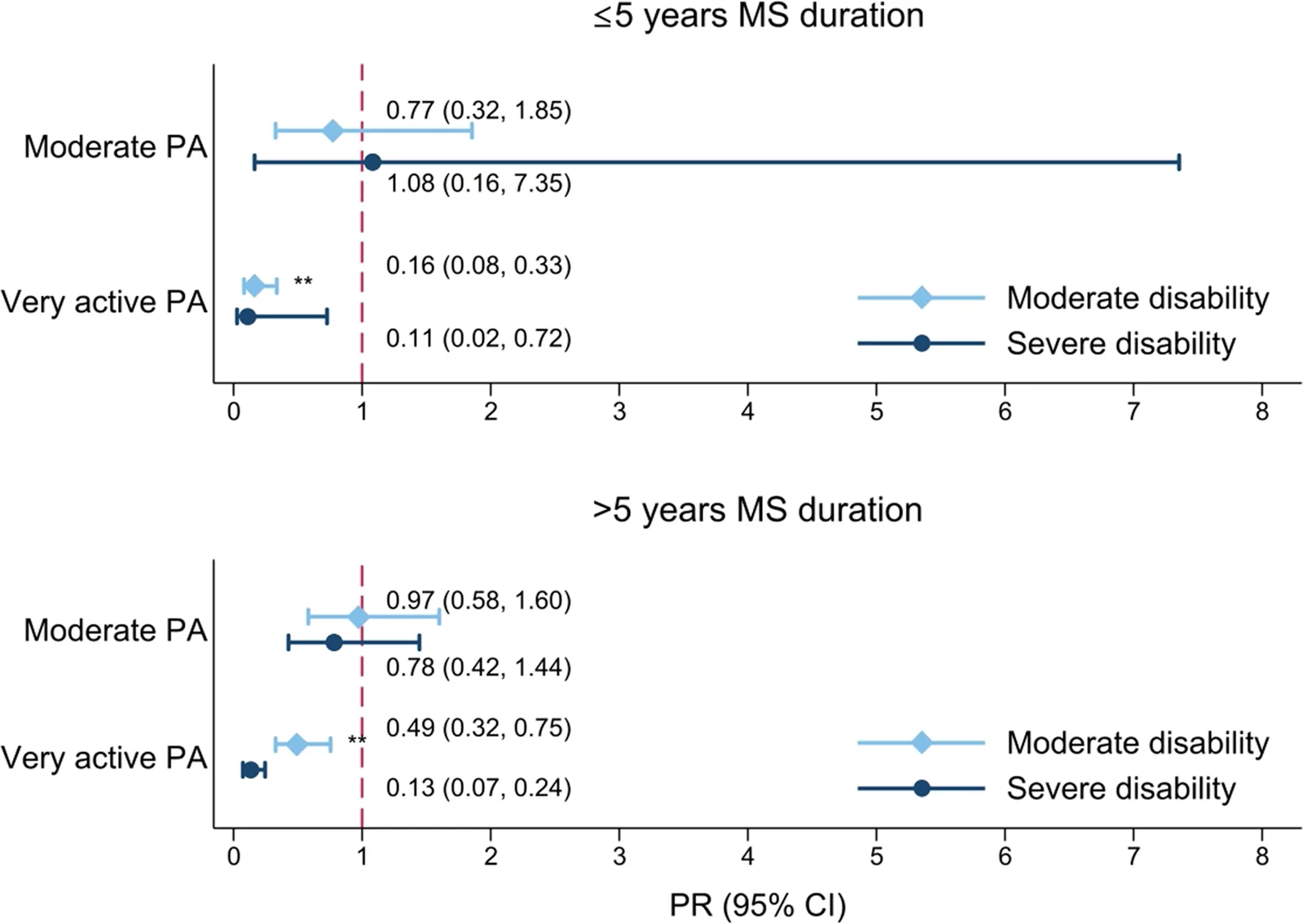
**Associations between physical activity and ambulatory disability by MS duration.** Note: MS = multiple sclerosis; PA = physical activity. ** indicates effect modification by MS duration for the association between very active physical activity and moderate ambulatory disability (PR _interaction_ = 3.06; 95% CI: 1.34–7.00; *P*_interaction_ = 0.008). Multinomial regression estimating adjusted relative prevalence ratios (95% CI). Models adjusted for age, sex, body mass index, education, symptoms before diagnosis, and MS phenotype. Error bars represent 95% CIs; associations were considered statistically significant if the 95% CI did not include 1.0

Diet, wellness activities, and supplement use were not associated with ambulatory disability in either duration group (data not shown). Physical activity was consistently associated with lower ambulatory disability prevalence in both MS duration groups (Fig. 1). Very active physical activity was associated with substantially lower prevalence of both moderate and high ambulatory disability relative to low ambulatory disability. These associations were observed in both duration groups, with evidence of effect modification by MS duration for moderate ambulatory disability (*P*_interaction_ =0.008).

## Discussion

In this cross-sectional study, physical activity showed the most consistent associations with better QoL and lower ambulatory disability in pwMS. In particular, very active physical activity was associated with better QoL across multiple domains and with lower prevalence of moderate disability, with some evidence that these associations were stronger in pwMS with ≤ 5-years MS duration. In contrast, associations for diet, wellness activities, and omega-3 supplement use were less consistent. Given the multidimensional nature of lifestyle factors examined, and potential for reverse causation, the findings are interpreted as exploratory, and need to be validated in future prospective studies with additional consideration of adherence to specific lifestyles, and at what point they were implemented.

Diet patterns showed limited associations with QoL in pwMS with ≤ 5-years MS duration. In contrast, diet patterns were more strongly associated with poorer QoL domain scores, particularly higher stigma and lower physical function, in pwMS with > 5-years duration. Specifically, low-saturated fat and other diet patterns (e.g., organic/low sodium) were associated with higher stigma in the > 5-years group, and other diets were also associated with lower fine motor. It is possible that adopting more restrictive diets is linked with greater perceived stigma over time; however, these cross-sectional findings may reflect reverse causation and warrant confirmation in longitudinal analyses.

Of wellness activity categories, mind activities were associated with lower QoL across multiple domains in pwMS with >5-years MS duration, and no associations for those with ≤5-years MS duration. However, there was no effect modification by MS duration. This contrasts with trials showing mindfulness and cognitive behavioural therapy improve depression, anxiety, and QoL [15, 30]. Our findings are likely reflective of reverse causation, whereby pwMS experiencing greater symptom burden may be more likely to engage in mind-based practices. Alternatively, the limitations of the survey querying use of multiple activities in the past six months rather than adherence to a single practice, as well as consolidation of a practices for analysis, may have masked associations specific types of activities. Other types of wellness activities were not associated with QoL or disability in either duration group. Recent reviews of mind-body (e.g., tai chi/qigong) and body-based therapies (e.g., massage/acupuncture) in MS generally report modest improvements in QoL, but emphasise heterogeneity, small trials, and variable certainty of evidence [31].

Of two supplements analysed, Vitamin D supplement use was not associated with QoL or disability, consistent with systematic reviews showing mixed or null effects depending on baseline deficiency and dosage [32, 33]. For MS management, vitamin D checks should be a routine occurrence and supplement use only recommended if deficient. Omega-3 supplement use was associated with improved mobility, fine motor function, sleep, and social satisfaction, with stronger associations in ≤5-years MS duration. However, these associations did not remain statistically significant after Bonferroni correction in sensitivity analyses therefore findings are interpreted with caution. While effect sizes were small, the findings extend prior evidence suggesting benefits of omega-3 for relapses, inflammation, and QoL [13], and support further evaluation in longitudinal studies. Clinical trials of omega-3/PUFAs in MS have generally been small and heterogenous and have shown no consistent improvement in disability outcomes [34]; in this cross-sectional analysis we did not observe an association with ambulatory disability.

Physical activity was consistently associated with better QoL and lower ambulatory disability. Moderate activity was linked with lower depression scores in pwMS with >5-years MS duration, while very active levels was associated with better outcomes in nearly all QoL domains across both MS duration groups. Very active physical activity was also associated with lower levels of moderate and severe ambulatory disability, with effects strongest in ≤ 5-years MS duration. These findings align with prior evidence that regular physical activity improves fatigue, mobility, and psychological wellbeing [10, 35], and highlight physical activity as a central component of MS management throughout the disease course.

Several limitations should be noted. First, the cross-sectional design, which while prevents causal inference informs future prospective research. It is possible that pwMS with greater symptom burden or poorer QoL may be more likely to adopt lifestyle practices to manage symptoms. Second, data capture, including self-reported lifestyle measures which may introduce recall bias, inherent for all survey-based studies. Data capture of lifestyle practices was queried as having used in the past six months not accounting for current practice nor duration or adherence to these practices. Multiple selections of any of over 20 practices and consolidation into broad categories as well as responses of used/used and helpful for analysis, potentially masked effects. Third, MS duration was defined as date from diagnosis, however symptom onset was a median of 7 and 20 years among ≤5-years and >5-years groups respectively, impacting disease progression; to account for this we adjusted regression models for symptoms prior to diagnosis. Finally, participants were a self-selected volunteer population, which may limit generalizability to pwMS engaged and interested in lifestyle practices for MS management.

Despite limitations, our study uniquely shows several associations between lifestyle and outcomes differing based on MS duration. Lifestyle factors, especially physical activity, was associated with higher QoL in pwMS, with the strongest effects seen within ≤5-years MS duration. Physical activity is also associated with reduced ambulatory disability levels regardless of MS duration. These findings underscore the importance of integrating lifestyle counselling early and longitudinally within MS care pathways, alongside pharmacological management.

Future prospective studies assessing individual and multimodal lifestyle practices with comprehensive measurements of lifestyle practices, along with mechanistic studies to understand pathways linking lifestyle with neuroprotection and symptom management, are needed. Additionally, studies focusing on how best to integrate lifestyle practices into MS management while addressing barriers to adoption and adherence to such practices, are recommended.

## Supplementary Information

Below is the link to the electronic supplementary material.

Supplementary Material 1

## Data Availability

De-identified raw data was purchased from Accelerated Cure Project for Multiple Sclerosis upon approval of the study proposal by the iConquerMS Research Committee. Aggregate data for the current study may be requested from NN.
